# The Effect of Eye Movement Desensitization and Reprocessing (EMDR) Therapy on Reducing Craving in Populations with Substance Use Disorder: A Meta-Analysis

**DOI:** 10.3390/brainsci14111110

**Published:** 2024-10-31

**Authors:** Diana Emilia Martínez-Fernández, David Fernández-Quezada, Andrea P. Garzón-Partida, Irene G. Aguilar-García, Joaquín García-Estrada, Sonia Luquin

**Affiliations:** 1 Instituto Transdisciplinar de Investigación y Servicios (ITRANS), Universidad de Guadalajara (UdeG), Zapopan 45150, Mexico; diana.martinez@academicos.udg.mx; 2 Departamento de Farmacobiología, Centro Universitario de Ciencias Exactas e Ingenierías, Universidad de Guadalajara (UdeG), Blvd. Marcelino García Barragán, Guadalajara 1421, Mexico; 3 Departamento de Neurociencias, Centro Universitario de Ciencias de la Salud (CUCS), Universidad de Guadalajara (UdeG), Sierra Nevada 950, Guadalajara 44340, Mexico; 4 Doctorado en Ciencias Biomédicas, Centro Universitario de Ciencias de la Salud (CUCS), Universidad de Guadalajara (UdeG), Guadalajara 44340, Mexico; 5 Maestría en Neurociencias de las Adicciones, Centro Universitario de Ciencias de la Salud (CUCS), Universidad de Guadalajara (UdeG), Guadalajara 44340, Mexico

**Keywords:** EMDR, craving, substance use disorder, addiction, meta-analysis

## Abstract

Substance use disorder (SUD) significantly impacts public health, economics, and legal systems worldwide. Eye Movement Desensitization and Reprocessing (EMDR) was initially developed in the late 1980s as a therapeutic approach for post-traumatic stress disorder (PTSD), using bilateral stimulation to integrate traumatic memories with calming physiological responses. However, the effectiveness of EMDR in treating SUD remains unclear. This study aims to conduct a systematic review and meta-analysis of the impact of EMDR therapy on craving reduction in individuals with SUD. The search was conducted using databases such as PubMed and Web of Science, focusing on studies that measured craving and employed EMDR interventions. Both random and fixed effects models were used to pool effect sizes, utilizing an R software meta-package (R-4.4.1). The study adhered to the Preferred Reporting Items for Systematic Reviews and Meta-Analyses (PRISMA) guidelines. The results indicated a significant reduction in cravings among patients undergoing EMDR therapy. Specifically, under the fixed effect model, the standardized mean difference (SMD) was −0.866 with a 95% confidence interval ranging from −1.121 to −0.611 (z = −6.66, *p* < 0.0001). These findings may demonstrate the significant efficacy of EMDR therapy in decreasing cravings in people with SUD.

## 1. Introduction

Substance use disorder (SUD) exhibits considerable prevalence that fluctuates based on geographical regions [1]. The effects of SUD on community health, economic conditions, and legal structures are considerable and worsening [2]. SUDs lead to heightened mortality rates, drain economic resources, and challenge legal systems [3]. Approximately 270 million individuals, or about 5.5% of the global population aged 15–64, consumed psychoactive substances in the past year, with an estimated 35 million people suffering from drug use disorders, which include harmful patterns of use or dependency. Annually, drug use is linked to roughly 500,000 deaths, divided between 350,000 men and 150,000 women. In recent times, deaths related to opioid use, primarily synthetic opioids, have altered mortality patterns in several high-income nations [4]. In 2017, drug use was responsible for the loss of over 42 million years of healthy life, accounting for approximately 1.3% of the global disease burden [4]. In México, eight out of ten adolescents, either in detention centers or subject to external corrective measures, reported having used some type of psychoactive substance at some point in their lives, with alcohol, tobacco, and marijuana being the most prevalent [5]. Particularly, there has been a surge in drug-related overdose deaths, which is a trend that has sharply increased over the past decade and intensified during the COVID-19 pandemic [6].

Addiction is described as a progressive cycle consisting of three stages: binge/intoxication, withdrawal/negative affect, and preoccupation/anticipation. This cycle involves changes in the brain’s reward and stress systems [7]. The cycle is driven by two types of reinforcement: positive reinforcement, which encourages substance use through pleasurable effects, and negative reinforcement, which motivates use by reducing or avoiding negative feelings [8,9]. The chronic administration of drugs leads to increased reward thresholds during withdrawal, effectively reducing the perceived reward from drug use. A major challenge in drug addiction is chronic relapse, where individuals repeatedly revert to compulsive drug use long after the acute withdrawal symptoms have subsided. Persistent withdrawal can lead to intense cravings [7,10].

A crucial element of SUD is the development of persistent craving, which is defined as a compelling and intrusive desire or compulsion to use a drug. This craving is primarily driven by memories of the drug’s rewarding effects, which are closely associated with a negative emotional state [7,11,12]. It is suggested that cravings, triggered by cues linked to drug use, progressively intensify during the early stages of abstinence and continue to be elevated over prolonged periods [11]. Research indicates that maladaptive memory associations contribute to the perpetuation of behaviors linked with substance abuse and the experience of cravings. These associations often reinforce negative patterns of behavior, making the cycle of addiction more difficult to break [9,13]. Cravings are often sustained and intensified by vivid sensory imagery stored in memories, where more intense imagery is associated with stronger cravings. Craving is a significant factor in addictive behaviors [11,14], and substantial evidence [15] supports its classification as one of the primary criteria for diagnosing substance use disorders (SUDs) in the most recent edition of the Diagnostic and Statistical Manual of Mental Disorders (DSM-5).

The presence of craving and deficits in inhibitory control are considered crucial in addiction particularly when individuals encounter specific behavioral triggers [12]. Moreover, findings suggest that alleviating cravings is linked to subsequent reductions in substance use and its associated negative consequences. Consequently, craving is regarded as a potential predictor of relapse and may discourage individuals from attempting to cease their substance use [12,16].

Eye Movement Desensitization and Reprocessing (EMDR) was developed in the late 1980s as a psychological treatment for post-traumatic stress disorder (PTSD) [17], although its clinical applications have been extended considerably over the years. EMDR can mitigate the vividness of these memory images through reprocessing techniques, presenting a viable therapeutic option for addressing such cravings [13]. The Adaptive Information Processing (AIP) model is the prevailing theory used to explain the mechanisms behind Eye Movement Desensitization and Reprocessing (EMDR). This model posits that there is an inherent system within everyone that is physiologically designed to process information toward a state of mental health. It is believed that EMDR leverages bilateral stimulation to facilitate the connection and reintegration of traumatic experiences with relaxing physiological responses [18,19]. Additional theories propose that Eye Movement Desensitization and Reprocessing (EMDR) therapy may enhance interhemispheric interaction, mimic the effects of Rapid Eye Movement (REM) sleep, or fatigue the working memory. These mechanisms are thought to contribute to alterations in the recall of traumatic memories, aiding in their processing and the reduction in trauma-related symptoms [20,21,22,23,24,25,26]. However, the precise mechanisms behind its efficacy remain inconclusive. Recently, Baek et al. reported that EMDR can reduce amygdala activation [27], and De Jongh et al. support that the bilateral stimulation used in EMDR sends inhibitory signals to the amygdala, potentially mitigating emotional responses [28]. Recently, our laboratory presented the first histological report detailing the effects of EMDR. Our findings indicate that visual EMDR stimulation could mitigate hippocampal atrophy during exposure to acute variable stress [29].

Despite its potential, the feasibility of implementing EMDR among individuals with SUD has not been thoroughly researched, and findings regarding its effectiveness are still unclear. Consequently, the utility of EMDR sessions in reducing cravings among this population remains questionable. Therefore, the objective of this study was to assess the research that has integrated EMDR to determine its effectiveness in reducing cravings and to explore the relationship between EMDR therapy and craving reduction in individuals with SUD.

## 2. Materials and Methods

The meta-analysis was developed based on the PROSPERO (CRD42024531371) on 23 April 2024. The search strategy for each database was developed in accordance with the research question, adhering to the Preferred Reporting Items for Systematic Reviews and Meta-Analyses (PRISMA) guidelines [30] and WorkBooks of Meta-Essentials [31].

### 2.1. Search Strategy

They were collected from previously published studies that obtained approval from ethical committees. Two independent authors (D.E.M.-F., A.P.G.-P.) searched the Web of Science and PubMed from database inception to 1 February 2024. The search strategy was crafted using a combination of Medical Subject Headings (MeSH) terms and various free-text keywords. Additionally, we enhanced our electronic database searches by reviewing reference lists from pertinent review articles and implemented a forward citation-tracking process for key papers in the field. The primary search was to identify articles that had used EMDR to treat patients with substance use disorder, specifically craving. Therefore, the search key words used were based on terms related to “EMDR” AND “Addiction” OR “Addicted” OR “Substance use” OR “Substance abuse”, OR “Drug” in the title, abstract or index term fields. The detailed search is shown in Table 1.

### 2.2. Inclusion and Exclusion Criteria

We included all studies that evaluated the effect of EMDR on individual with SUD and assessed craving levels in the meta-analysis. Only studies reporting measurable quantitative outcomes, such as performance measures or tests, were considered. If the abstract lacked sufficient information for inclusion, the full text was reviewed. The analysis was restricted to studies published in English. Exclusions included letters to the editor, clinical cases, commentaries, systematic reviews, qualitative studies, short communications, and meta-analyses. Additionally, if the mean and standard deviation were missing from a graph or table, attempts were made to contact the authors for the necessary data. Any full-text publications that were inaccessible, and for which no response was obtained from the corresponding author after two email attempts, were also excluded; see Table 2.

### 2.3. Data Extraction

**Figure 1:**
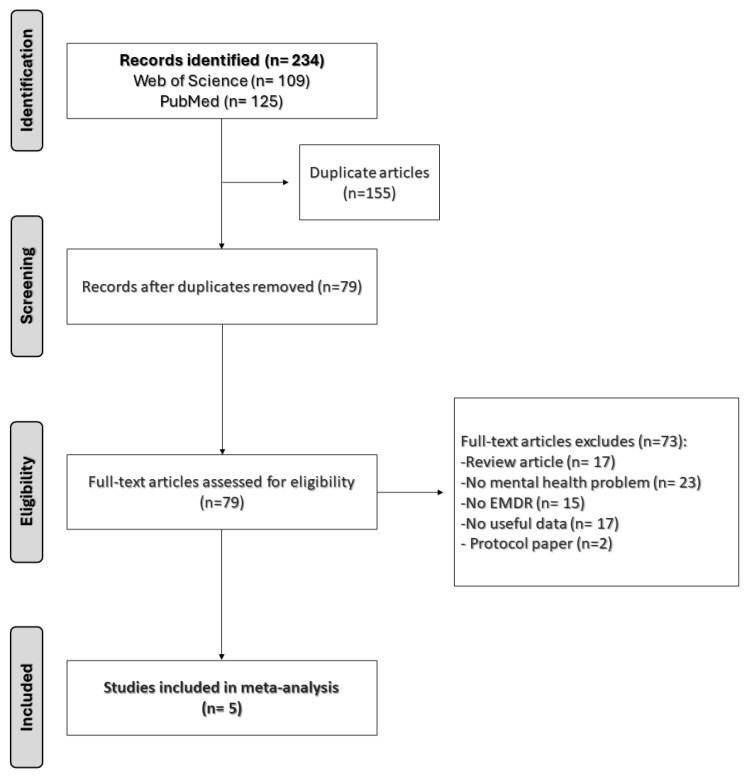
Flow diagram of study selection process [32,33,34,35].

Two authors (D.E.M.-F., A.P.G.-P.) independently screened and identified eligible papers by title, abstract, and full text against prespecified criteria. After the selection process (see Figure 1), relevant data from the selected studies were extracted to an Excel sheet. The researchers independently coded the studies for the variables of interest: first author, year, publication venue, design classification, total sample size, sample size for each group and measure, sample age, primary measure, study location by country, treatment description, number of EMDR sessions, and effect size information. Finally, the effect size calculations were performed by a single researcher in collaboration with the other two researchers who provided input and guidance on decisions when the information presented in the studies was unclear or ambiguous.

### 2.4. Risk of Bias-2

The assessment of risk of bias (RoB-2) was conducted by two independent researchers, I.G.A.-G. and J.G.-E., with a third researcher, D.F.-Q., available for mediation if needed. However, mediation was unnecessary due to consistent agreement among the reviewers. The authors utilized the Cochrane Risk of Bias-2 (RoB-2) tool [36] to evaluate the quality of the research. This tool assesses risk across five distinct domains: (1) randomization process, (2) deviations from the planned intervention, (3) missing outcome data, (4) outcome measurement, and (5) selection of reported results. Each domain is rated on three levels of risk: high, some concerns, and low, which is based on signaling questions within that domain. The overall risk of bias for the study is determined by integrating the evaluations from these domains to form a comprehensive view. A study is typically classified as having a high risk of bias if any domain is rated as high risk or if multiple domains are assessed as having some concerns. Conversely, a study is considered to have a low risk of bias if all domains are rated as low risk. If only a few domains have some concerns, the study is regarded as having some concerns overall.

### 2.5. Data Analysis

Both random and fixed effects models were used to pool the estimate of the EMDR effects on craving in individuals with SUD. To calculate pooled effect sizes, an R language (version 4.1.2, http://www.Rproject.org, accessed on 1 April 2024) R “meta” package was employed (Supplementary S1: analytic code in R). We calculated the mean difference (MD), and then the pooled SD is calculated to reflect the shared variability between the groups, while the standard error (SE) is obtained by multiplying the pooled SD by the square root of the sum of the inverse of the sample sizes from each group. The standardized mean difference (SMD) is determined by dividing the MD by the pooled SD, and it represents Cohen’s d. Given the tendency of small sample sizes to overestimate the overall effect size, Hedges’ g is applied as an adjustment to provide a more accurate estimation [37]; see Table 3. The I^2^ statistic was used to measure the degree of heterogeneity caused by variability in the true effect size. The effect sizes of g = 0.20 are small, g = 0.50 as moderate and g = 0.80 as large [28]. Forest plots were created by the meta, metadata, metafor and rmeta function of meta packages, and funnel plots were constructed by the funnel function to estimate the publication bias.

## 3. Results

In this meta-analysis, we evaluated the efficacy of Eye Movement Desensitization and Reprocessing therapy on craving reduction in patients with substance use disorder. The strategy search identified 234 records, and the analysis incorporated findings from five studies, which were uniformly distributed between experimental (134 participants) and control groups (132 participants). The PRISMA diagram in Figure 1 shows the study selection process for this study.

### 3.1. Study Characteristics

The studies were published between 2008 and 2023 with a sample size ranging from 30 to 109 participants in each study. The samples were aged between 18 and 59 years old with the average age of patients ranging from 23.4 to 47.1. The studies included an active control group with distinct types of psychotherapy including cognitive behavioral therapy (CBT), eye movement (EM), exposure, and finger tapping. The application time for EMDR in all studies varied widely from 24 s to 1.5 h for each session or set, up to twice a week, and the total amount of sessions ranged from two to seven sessions. Craving was assessed in the studies included in this meta-analysis using several validated instruments. Specifically, the Obsessive-Compulsive Drinking Scale (OCDS) was utilized to evaluate obsessive thoughts and compulsive behaviors related to alcohol consumption, offering insights into the cognitive dimensions of craving. The Visual Analog Scale (VAS) was employed, enabling participants to rate the intensity of their craving on a continuum, which facilitated a straightforward quantification of their subjective experiences. Additionally, the Penn Alcohol Craving Scale (PACS) was incorporated to capture various dimensions of alcohol craving, focusing on both its frequency and intensity. Lastly, the Brief Substance Craving Scale (BSCS) was used for its efficiency in measuring overall craving for substances. The characteristics of the selected studies are summarized and presented in Table 4.

### 3.2. Effect of EMDR Therapy on Craving Reduction in Substance Use Disorder

The results revealed a significant reduction in craving among patients receiving EMDR therapy. Specifically, under the common effect model, the SMD was −0.866 with a 95% confidence interval ranging from −1.121 to −0.611 (z = −6.66, *p* < 0.0001). The random effects model, which accounts for variability among studies, indicated a slightly larger effect size (SMD = −0.987) with a 95% confidence interval from −1.410 to −0.564 (z = −4.57, *p* < 0.0001).

**Figure 2:**
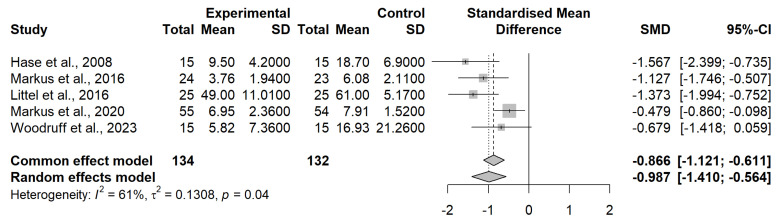
Forest plot. Negative values indicate less craving symptoms in the EMDR intervention than the control condition.

Regarding the heterogeneity among the included studies, it was found to be moderate to high, I^2^ of 60.7%, indicating a moderate to high variation in effect sizes. The tau2 value was 0.3616, indicating moderate between-study variance, while the heterogeneity test (Q = 10.17, df = 4, I2 = 60.7%, *p* = 0.0376) was statistical significantly, suggesting that the observed variability could be attributed to the studies, as shown in Figure 2.

### 3.3. Sensitivity Analysis

A sensitivity analysis was conducted sequentially excluding each individual study to evaluate their impact on the combined effect size. The findings indicated that the overall effect size remained relatively stable with individual study effect sizes varying between SMD = −0.7633 and −1.1805. This consistency supports the robustness and dependability of the meta-analysis results.

### 3.4. Publication Bias

**Figure 3:**
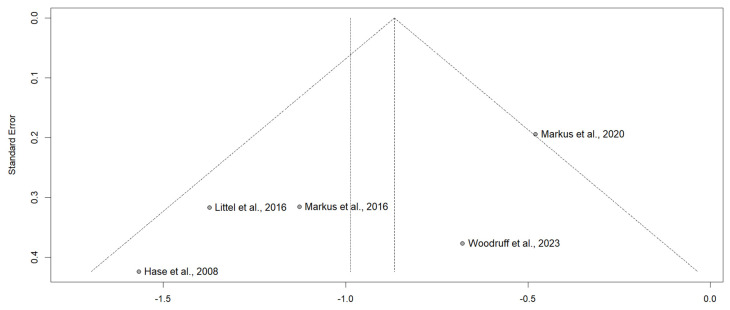
Funnel plot.

**Figure 4:**
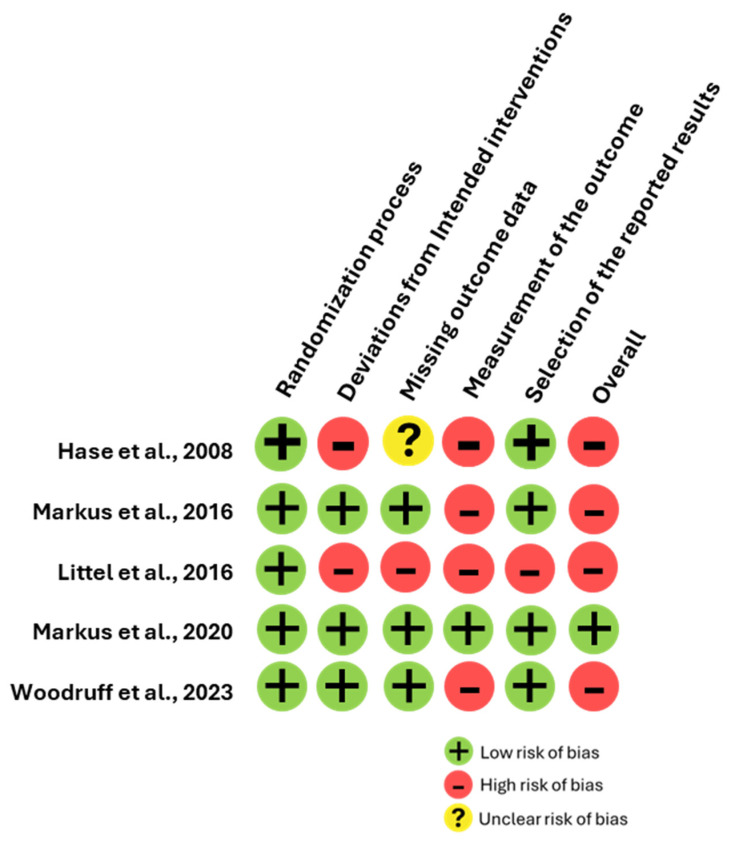
Cochrane Risk-of-Bias tool for randomized trials version 2 (RoB-2). In this color-coded ranking, green color represents low risk of bias, yellow unclear risk of bias, and red high risk of bias.

The funnel plot analysis revealed three studies positioned in the lower left quadrant and two studies in the right quadrant, suggesting some degree of symmetry; see Figure 3. However, given the small sample size of only five studies and their moderate heterogeneity, the risk of false positives in the meta-analysis is elevated, which can impact the interpretation of the funnel plot’s symmetry. Furthermore, the Rob-2 assessment indicated that most studies have a high risk of bias, which adds another layer of complexity to the findings; see Figure 4.

## 4. Discussion

A total of 266 participants were included in this meta-analysis to determine the effectiveness of EMDR on craving reduction in patients with substance use disorder. The results indicate that EMDR therapy significantly reduces craving compared with control conditions. The overall effects size indicates that EMDR therapy had a significant effect on decreasing craving. However, there was a significant moderate heterogeneity among the included studies indicating that the effect of EMDR on craving reduction may vary depending on study-specific factors. Therefore, these results should be interpreted with some caution.

While EMDR appears to be generally effective in reducing cravings, the variability between studies suggests that the magnitude of this effect might differ across different contexts or populations. This level of heterogeneity could stem from differences in study design, participant characteristics, or variations in how cravings were measured across studies. For instance, slight variations in the implementation of EMDR protocols or differences in the severity of substance use disorders among participants could contribute to this variability. Additionally, the number of studies included (n = 5) is relatively small, which can sometimes lead to an overestimation of heterogeneity. The moderate level of heterogeneity does not undermine the overall findings but highlights the need for future studies to explore these differences more thoroughly.

Although the funnel plot did not indicate significant publication bias, it is important to recognize that the absence of visual asymmetry does not entirely eliminate the possibility of such bias. However, publication bias may still exist, particularly if smaller studies with non-significant or negative results are underreported or excluded from publication, potentially distorting the overall evidence. This is why the RoB-2 assessment indicated that most studies have a high risk of bias. Also, in research fields where positive findings are more frequently published, there is a possibility that the perceived impact of EMDR on craving reduction could be overstated. While the funnel plot provides some reassurance, caution should be exercised in interpreting these results. The potential omission of studies with null or negative findings suggests that the true effect size may be smaller than our analysis indicates. Consequently, it is essential for future research to address this issue by ensuring a comprehensive reporting of all study outcomes regardless of their statistical significance.

Furthermore, the beneficial effects of EMDR are primarily linked to bilateral sensory stimulation, which is typically triggered by the therapist’s hand movements that prompt lateral eye movements in patients as they recall distressing memories. This form of bilateral stimulation is thought to enhance information processing and promote an adaptive consolidation of memories. Consequently, it helps diminish emotional distress and promote psychological resilience. This mechanism is believed to underlie the effectiveness of EMDR in alleviating psychological stress and enhancing mental health recovery [38,39,40]. Interestingly, EMDR therapy has been shown to diminish both the vividness and the emotional intensity of positively charged memories [41,42] and even images of potential future events (flash-forwards) [43,44,45], suggesting that EMDR could be effectively used to treat a range of psychopathologies where maladaptive memories and mental imagery are significant factors, such as in cases of substance abuse disorders. The ability of EMDR to modulate the emotional response associated with both negative and positive memories indicates its potential as a versatile therapeutic tool in diverse clinical settings.

In addictive disorders, the act of recalling substance-related memories plays a crucial role in triggering cravings. These cravings are a significant factor in the continuation of substance use and notably increase the risk of relapse. The vividness and emotional intensity of these memories can strongly influence an individual’s ongoing struggle with addiction [46,47]. These memories include both classical and instrumental associations, for example, the links between stress and smoking or smoking and relaxation as well as episodic memories. Episodic memories in this context might involve recalling the first time a substance was used, the consequences of substance use, and experiences related to losing control or relapsing. This comprehensive memory framework plays a pivotal role in how cravings are triggered and managed, impacting the cycle of addiction and recovery efforts [48,49]. Cravings are frequently intensified by sensory imagery, which may involve visualizing the substance, imagining its smell, or anticipating future use. This type of vivid mental imagery can make the craving more acute, effectively reinforcing the desire to use the substance and making it harder for individuals to resist the impulse to relapse [47,50].

Craving reduction can be effectively facilitated through dual-task procedures. Research indicates that when individuals engage in imagery or visuospatial tasks that are unrelated to substance use during moments of intense craving, there is a significant decrease in both the frequency and intensity of these cravings. This method distracts the brain, diverting attention from substance-related thoughts and reducing the overall craving experience [51]. This concurrent cognitive activity serves as an effective coping mechanism to manage the acute effects of cravings and can be seamlessly integrated into clinical practice. To implement this method, individuals experiencing cravings are encouraged to engage in a dual task [52]. This could involve simultaneously performing a cognitive task that requires attention, such as solving a puzzle or engaging in a memory game, while experiencing craving episodes. This distraction helps mitigate the intensity of the cravings by occupying the mind with alternative stimuli, reducing the focus on the desire to use the substance. However, the effectiveness of this approach hinges on the ability of individuals with substance dependencies to recognize the onset of cravings while they are still manageable. Early recognition allows for timely intervention with dual-task activities, preventing cravings from escalating to a level that is more challenging to control [53]. In addition, EMDR treatment has been observed to positively influence emotion regulation. As a result, it facilitates the upregulation of positive and desirable emotions, making them more readily expressed [38].

Research on the full application of EMDR therapy specifically for addiction treatment remains limited. Most studies have primarily concentrated on the use of EMDR to address traumatic memories in individuals who have comorbid PTSD [54,55] and not on memory representations or sensory imagery constituting substance craving and dependence itself [56]. Despite a considerable volume of literature on EMDR and dependency disorders, most of these publications are case studies, proposed protocols for future research, theoretical discussions, or studies that do not specifically focus on substance use disorders. This indicates that while there is growing interest and theoretical backing for using EMDR in the context of dependency, there is a scarcity of rigorous empirical research directly investigating its effectiveness in treating substance use disorders. This lack of focused research underscores the need for well-designed studies to evaluate EMDR’s efficacy specifically in the realm of addiction treatment.

Future research should focus on larger sample sizes and extended follow-up periods to enhance the quality of evidence regarding the long-term benefits of EMDR for individuals with SUD. It is crucial to minimize bias by ensuring rigorous randomization procedures and conducting pretest equivalence checks between groups. Additionally, maintaining robust sample sizes is essential, particularly as follow-up assessments often see reduced participation. Researchers should account for potential attrition by planning for adequate sample sizes and implementing strategies to sustain participant involvement throughout the study. Furthermore, it is important for researchers to thoroughly report potential moderating or confounding variables, including demographic factors (e.g., age, gender, ethnicity) and methodological details (e.g., baseline assessments, measurement reliabilities, specific memory types addressed, treatment duration, EMDR protocols, and any adaptations).

## 5. Conclusions

These findings suggest that EMDR therapy may have potential benefits in reducing cravings among individuals with substance abuse disorders. However, it is important to acknowledge several limitations in interpreting these results. Variability in study designs and differences in participant demographics could impact the generalizability of our findings. Additionally, the small number of studies included in the analysis limits the strength of our conclusions. These factors highlight the need for further research with larger and more diverse populations to better understand and confirm the effectiveness of EMDR in treating substance abuse disorders.

## Figures and Tables

**Table 1:** Comprehensive search strategy for articles on EMDR treatment of craving in patients with substance use disorder.

Database	Search Strategy	Number of Results
PubMed	# (“eye movement desensitization reprocessing”[MeSH Terms] OR (“eye”[All Fields] AND “movement”[All Fields] AND “desensitization”[All Fields] AND “reprocessing”[All Fields]) OR “eye movement desensitization reprocessing”[All Fields] OR “emdr”[All Fields]) AND (“addict”[All Fields] OR “addict s”[All Fields] OR “addicted”[All Fields] OR “addicting”[All Fields] OR “addiction s”[All Fields] OR “addictive”[All Fields] OR “addictiveness”[All Fields] OR “addictives”[All Fields] OR “addicts”[All Fields] OR “behavior, addictive”[MeSH Terms] OR (“behavior”[All Fields] AND “addictive”[All Fields]) OR “addictive behavior”[All Fields] OR “addiction”[All Fields] OR “addictions”[All Fields] OR (“addict”[All Fields] OR “addict s”[All Fields] OR “addicted”[All Fields] OR “addicting”[All Fields] OR “addiction s”[All Fields] OR “addictive”[All Fields] OR “addictiveness”[All Fields] OR “addictives”[All Fields] OR “addicts”[All Fields] OR “behavior, addictive”[MeSH Terms] OR (“behavior”[All Fields] AND “addictive”[All Fields]) OR “addictive behavior”[All Fields] OR “addiction”[All Fields] OR “addictions”[All Fields]) OR (“substance related disorders”[MeSH Terms] OR (“substance related”[All Fields] AND “disorders”[All Fields]) OR “substance related disorders”[All Fields] OR “substance”[All Fields] OR “substance use”[All Fields]) OR (“substance related disorders”[MeSH Terms] OR (“substance related”[All Fields] AND “disorders”[All Fields]) OR “substance related disorders”[All Fields] OR (“substance”[All Fields] AND “abuse”[All Fields]) OR “substance abuse”[All Fields]) OR “Drug”[All Fields])	125 (26 February 2024)
Web of Science	# TS = (“EMDR”) AND TS = ((“addiction” OR “addicted” OR “substance use” OR “substance abuse” OR “drug abuse” OR “chemical dependency”))	109 (26 February 2024)

**Table 2:** Summary of studies excluded from the meta-analysis assessing the impact of EMDR therapy on craving in substance use disorder populations.

Reasons	Reference
Review article (*n* = 17)	(Buhmann et al., 2017); (Rens et al., 2012); (Bahji et al., 2022); (Dansiger et al., 2020); (Besson et al., 2006); (Luteijin et al., 2020); (MacGuire et al., 2014); (Sonis et al., 2019); (Mamtani et al., 2023); (Modlin et al., 2024); (Knorring et al., 2005); (Finnegan et al., 2016); (Ronconi et al., 2014); (Carlson, 2005); (Watts et al., 2013); (Foa, 2000); (O’Shea, 2001).
No mental health problem (*n* = 23)	(Moreno-Alcázar et al., 2017); (van den Berg et al., 2016); (Arnone et al., 2012); (Bossinni et al., 2012); (Minelli et al., 2019); (Park et al., 2016); (Torricelli et al., 2021); (Omeragic et al., 2021); (Kapfhammer et al., 2008); (Mavranezouli et al., 2020); (Chendamarai et al., 2015); (Hofel et al., 2018); (Forbes et al., 2007); (Merians et al., 2023); (Babaei et al., 2023); (Arntz et al., 2022); (Bae et al., 2018); (Ipci et al., 2017); (Sahpiro et al., 2013); (van der Kolk et al., 2007); (Manzoni et al., 2021); (Gielkens et al., 2016); (Yoshii, 2021).
No EMDR (*n* = 15)	(Bossini et al., 2017); (Feldhahn et al., 2012); (Shain et al., 2014); (Wen et al., 2017); (Lifshitz et al., 2017); (Oppermann-Schmid et al., 2022); (Ganesan et al., 2016); (Delgadillo et al., 2021); (Sanchez et al., 2019); (El-Barazi et al., 2023); (Suzuki et al., 2023); (Morgenthaler et al., 2018); (Aurora et al., 2010); (Sugimoto et al., 2015); (Uccellini et al., 2022).
No useful data (*n* = 17)	(Marich et al., 2010); (Ysebaert et al., 2011); (Colbert et al., 2024); (Worley et al., 2020); (Shapiro et al., 1994); (Palumbo et al., 2020); (jeal et al., 2018); (Patel et al., 2020); (Sedin Habibovi, 2021); (Carletto et al., 2018); (Alicia Valiente-Gómez et al., 2019); (Chen et al., 2019); (Meysami-Bonab et al., 2016); (Perez-Dandieu et al., 2014); (Bae et al., 2015); (ströhle et al., 2000); (Nejra Zejnullahu, 2021).
Protocol paper (*n* = 2)	(Markus et al., 2015); (Lortye et al., 2021).

**Table 3:** Detailed compilation of extracted data from studies researching EMDR therapy’s efficacy in reducing craving among substance use disorder patients.

Study	n1	m1	s1	n2	m2	s2	md	sd	se	cohen_d	cohen_se
Hase et al., 2008	15	9.5	4.2	15	18.7	6.9	−9.2	5.7118	2.0857	−1.6	0.4231
Markus et al., 2016	24	3.76	1.94	23	6.08	2.11	−2.32	2.0249	0.5894	−1.1	0.3139
Littel et al., 2016	25	49	11.01	25	61	5.17	−12	8.6008	2.4327	−1.4	0.3168
Markus et al., 2019	55	6.95	2.36	54	7.91	1.52	−0.96	1.9888	0.381	−0.5	0.1945
Woodruff et al., 2023	15	5.82	7.36	15	16.93	21.26	−11.11	15.9	5.8059	−0.7	0.3769

n1, EMDR group sample size; m1, EMDR group mean; s1, EMDR group standard deviation; n2, control group sample size; m2, control group mean; s2, control group standard deviation; md, mean difference; sd, pooled sd; se, pooled standard error; cohen_d, SMD; cohen_se, standard error of SMD; g, group.

**Table 4:** Summarized characteristics of the studies.

Study	Control Condition	EMDR Intervention	Intervention Duration	Country	Risk of	Study	Control Condition	EMDR Intervention
Hase et al., 2008	TAU	TAU + EMDR	2 sessions, 1 h, 1 week	Germany	High	30	45.7 (5.2)	OCDS
Markus et al., 2016	EM-	EM +	7 sessions, 1.5 h, 1 time/week	The Netherlands	High	47	32.13 (13.69)	VAS
Littel et al., 2016	RO	+ EM	6 sets, intervals of 24 s	The Netherlands	High	50	23.4 (6.6)	VAS
Markus et al., 2019	TAU	AF-EMDR	7 sessions, 1.5 h, 1 time/week	The Netherlands	Low	109	47.1 (11.6)	PACS
Woodruff et al., 2023	CBT	AF-EMDR + CBT	4 sessions, 1 h, twice a week	USA	High	30	40.07 (13.32)	BSCS

TAU, treatment as usual; EM-, equivalent control EMDR without Eye Movements; RO; recall only; EM+, experimental condition of EMDR procedure; AF-EMDR, addiction-focused eye movement desensitization reprocessing; CBT, cognitive behavioral therapy; OCDS, Obsessive-Compulsive Drinking Scale; VAS, Visual Analog Scale, PACS, Penn Alcohol Craving Scale; BSCS, Brief Substance Craving Scale.

## Data Availability

The data extracted from the included studies and the analytic code are available.

## Supplementary Materials

The following supporting information can be downloaded at https://www.mdpi.com/article/10.3390/brainsci14111110/s1, S1: analytic code in R.
