# Supplementary material for: The Effect of Eye Movement Desensitization and Reprocessing (EMDR) Therapy on Reducing Craving in Populations with Substance Use Disorder: A Meta-Analysis
Source: Brain Sci. 2024 Oct 31;14(11):1110. doi: 10.3390/brainsci14111110 (PMC11592247; doi:10.3390/brainsci14111110)
Supplement: Supplementary file 1 [file brainsci-14-01110-s001.zip › brainsci-3216123-supplementary.pdf]

Supplementary Material S1

R Code

```
#SMD

cont <- metacont (n1, m1, s1, n2, m2, s2, sm="SMD", method.smd="Hedges", studlab = study, data = cont); print(cont, digits =3);

#forest plot

forest(cont, comb.fixed=TRUE, comb.random=TRUE,digits=3,rightcols=c("effect", "ci"));

#metadata

metainf(cont);

#funnel plot

funnel(cont, comb.fixed=TRUE, comb.random=FALSE); text(x = cont$TE, y = cont$seTE, labels = cont$studlab, pos = 4, cex = 1.0)
```
